# Resilience and Psychobiological Response to Stress in Older People: The Mediating Role of Coping Strategies

**DOI:** 10.3389/fnagi.2021.632141

**Published:** 2021-02-22

**Authors:** Mariola Zapater-Fajarí, Isabel Crespo-Sanmiguel, Matias M. Pulopulos, Vanesa Hidalgo, Alicia Salvador

**Affiliations:** ^1^Laboratory of Cognitive Social Neuroscience, Department of Psychobiology and IDOCAL, University of Valencia, Valencia, Spain; ^2^Instituto de Investigación Sanitaria Aragón, Department of Psychology and Sociology, Area of Psychobiology, University of Zaragoza, Teruel, Spain

**Keywords:** resilience, coping, stress, cortisol, older people

## Abstract

Resilience, the ability to overcome adversity and face stressful demands and experiences, has been strongly associated with successful aging, a low risk of diseases and high mental and physical functioning. This relationship could be based on adaptive coping behaviors, but more research is needed to gain knowledge about the strategies employed to confront social stress. Thus, we aimed to investigate the role of the use of active or passive coping strategies by resilient people in dealing with stressful situations. For this purpose, we measured resilience, coping strategies, and perceived stress in 66 healthy older adults (31 men and 35 women) between 56 and 75 years old who were exposed to stress (Trier Social Stress Test, TSST) or a control situation. The stress response was analyzed at endocrine (cortisol) and psychological (anxiety) levels. In the stress condition, moderated mediation analysis showed a conditional indirect effect of resilience on cortisol reactivity through active coping. However, passive coping strategies did not mediate the resilience-cortisol relationship. In addition, neither active nor passive coping mediated the relationship between resilience and the anxiety response. These results suggest that resilience is associated with active coping strategies, which in turn could explain, at least in part, individual differences in the cortisol response to a psychosocial laboratory stressor. These factors may prevent the development of stress-related pathologies associated with aging and facilitate healthy and satisfactory aging.

## Introduction

Stress is considered one of the most significant health problems of the 21st century (World Health Organization, 2001), due to its contribution to numerous disorders, such as depression and sleep problems (Vos et al., 2016), and several age-related diseases (Zsoldos et al., 2014). Current research has focused on which personality or coping factors could explain individual differences in stress responsiveness, and thus provide an explanatory approach of the protective or vulnerability factors to the psychobiological effects of the stress response.

At a physiological level, the stress response is mainly characterized by the activation of two physiological systems: the autonomic nervous system, which increases blood pressure and heart rate (Allen et al., 2011), and the hypothalamic-pituitary-adrenocortical axis (HPA axis), with the consequent release of cortisol (McEwen, 2008). Furthermore, when exposed to stress, people experience emotional changes, such as increases in anxiety (Campbell and Ehlert, 2012; Villada et al., 2014a; Fan et al., 2015) and negative affect and decreases in positive affect (Schmaus et al., 2008; Villada et al., 2017). Moreover, over-reactivity of the HPA axis and heightened psychological reactivity (i.e., increased negative affect and anxiety) have been associated with increased risk of several health problems, such as cardiovascular disease, Type 2 diabetes, reduced immune function, and cognitive impairment (Kiecolt-Glaser et al., 2003; Lundberg, 2005). The allostatic load model explains the physiological effects underlying these conditions, given that a repeated or heightened HPA axis activation could led to the activation of the fight or flight response systems (i.e., cardiovascular, muscular, among others) resulting in an allostatic load, and thus multiple health problems (Sterling and Eyer, 1988; McEwen, 1998). Therefore, following the cortisol reactivity threshold model (Vrshek-Schallhorn et al., 2018), the more adaptive stress response would be characterized by moderated HPA axis activation, rather than increased HPA axis reactivity (Herman et al., 2016), and stable levels of negative emotions such as anxiety (Kiecolt-Glaser et al., 2002).

Much of this research, however, has focused on stress reactivity in young adults, even though older adults are more vulnerable to the chronic conditions associated with stress (for reviews, see: Uchino et al., 2005; Kudielka et al., 2009; Pulopulos et al., 2018). Studies with older adults have found that they tend to show a stronger cortisol response to stress and worse regulation of the HPA axis under stressful conditions (Pulopulos et al., 2018). Furthermore, most of the aforementioned health problems associated with stress have also been found to be related to the aging process (e.g., Type 2 diabetes or cognitive impairment) (Kiecolt-Glaser et al., 2003; Lundberg, 2005). Therefore, it is important to investigate the factors that can explain individual differences in the stress response of older people, in order to develop prevention programmes and intervention targets, and consider the role of the stress response in the development of health problems in this population (MacLeod et al., 2016).

There are important individual differences in the way individuals face stressful situations, which could determine their psychobiological response and, therefore, their risk of stress-related diseases. As Lazarus and Folkman (1984) indicated in their stress and coping model, individual differences in the stress response depend on both the subject's appraisal of the situation and the resources available to manage the stress. In this line, according to the broaden and build theory of positive emotions (Fredrickson, 1998), having a positive appraisal of the situation, by experiencing positive emotions even in stressful situations, broadens an individual's thought-action repertoire and may have the effect of enhancing his or her personal resources, including physical, intellectual, and social resources, thus promoting health and well-being.

In recent years, research has shown that resilience is strongly associated with an aging process characterized by low risk of disease and high mental and physical functioning (for a review on this topic, see MacLeod et al., 2016). Resilience is also considered a key factor in the optimal development of health and quality of life (Hjemdal et al., 2006; Souri and Hasanirad, 2011; Tomás et al., 2012). Resilience can be understood as an approach to managing stress that allows an individual to perceive a stressful situation as a challenge and engage in overcoming it (Connor and Davidson, 2003); resilient people experience growth and adaptation as well as recovery (Richardson, 2002). At a neurophysiological level, resilience promotes better regulation of the associations between the prefrontal cortex and hippocampus (Montes-Rodríguez and Urteaga-Urías, 2018) and enhanced control of the neural mechanisms of reward and motivation, fear responsiveness, and adaptive social behavior (Charney, 2004; Ryff, 2012).

With regard to the relationship between resilience and the stress response in older people, resilience has been suggested to modulate the association between diurnal cortisol and health by reducing alterations in daily cortisol patterns and, through this, promoting health (for review, see Gaffey et al., 2016). At a psychological level, resilience has been found to be related to positive emotions (Ong et al., 2006) and to reporting fewer adversities related to health and stress (Hildon et al., 2009). In contrast, low resilience has been associated with greater difficulties in regulating negative emotions and higher stress reactivity to daily stressors (Ong et al., 2006). To the best of our knowledge, no previous study has investigated the relationship between resilience and the psychobiological response to stressors in healthy older people. In this context, investigating the mechanisms that explain why resilient individuals have a more adaptive stress response may offer critical information to better understand inter-individual differences in stress regulation (Wu et al., 2013).

Coping strategies may be a mechanism through which resilient individuals face stressful demands and bounce back from negative experiences (Smith et al., 2015). However, several differences between resilience and coping should be acknowledged. Whereas, resilience influences how a stressful event is appraised, coping can be defined as the strategies (behavioral and cognitive) used to manage stressful demands after this appraisal. In addition, resilience seems to result in a positive response after a stressful situation, whereas coping can trigger both positive responses (e.g., active mobilization of sources) and negative responses (e.g., substance use) (Fletcher and Sarkar, 2013). In this regard, some authors have proposed that resilience promotes the active mobilization of resources under conditions of adversity. In other words, resilient individuals seem to employ adaptive coping strategies in stressful situations (Thompson et al., 2018). On the one hand, several studies have found that resilient older people use a more active coping style to manage adversity (for a review, see Southwick et al., 2005), and that resilience and active coping strategies play an important role in achieving well-being (Tomás et al., 2012; Mayordomo et al., 2016). In this regard, a study recently observed that postmenopausal females who actively coped with their condition (including engagement in planning strategies, acceptance or positive reinterpretation, and growth) showed better autonomic stress regulation (Villada et al., 2017). On the other hand, resilience also seems to be negatively related to avoidant (maladaptive) coping strategies (Hildon et al., 2009), and social withdrawal (an avoidant coping strategy) has been found to fully mediate the relationship between resilience and posttraumatic symptoms (Thompson et al., 2018). Given the strong relationship between resilience and coping and between coping and stress regulation, we expect older people with high resilience to develop active coping (adaptive) behaviors while reducing avoidance coping (maladaptive) behaviors in stressful situations, which in turn would enhance the regulation of the psychophysiological stress response.

Previous research shows the importance of resilience and coping strategies for health and well-being. The present study advances this research by systematically exploring the role of resilience and coping strategies in the stress response of older adults. The aims of this study were twofold: (1) to analyse the association between resilience and endocrine and psychological responses to acute stress in healthy older people; and (2) to investigate whether coping strategies mediate the relationship between resilience and the psychobiological response to stress. To examine this, we exposed 66 older adults to either the Trier Social Stress Test (TSST; Kirschbaum et al., 1993a) or a control situation, and measured their psychobiological stress responses in addition to resilience, coping strategies, and perceived stress.

Based on previous studies, we expected to find a negative association between resilience and cortisol and anxiety reactivity to a stressor (Mikolajczak et al., 2008; Ruiz-Robledillo et al., 2016) and a positive relationship between resilience and active coping (Thompson et al., 2018). At the same time, we expected that active coping would predict a more adaptive stress response characterized by moderated cortisol reactivity (Chida and Hamer, 2008; Villada et al., 2014b, 2017) and lower anxiety reactivity (Mahmoud et al., 2012; Villada et al., 2014b). In contrast, we expected that there would be a negative relationship between resilience and avoidance coping, and that less use of avoidance coping would predict a more adaptive stress response (Wu et al., 2013; Thompson et al., 2018). Finally, we expected that the effect of coping strategies on the relationship between resilience and the stress response would only be observed among participants in the stress condition, but not in the control condition.

## Materials and Methods

### Participants

Participants recruited for this study were part of a study programme for people aged over 55 years at the university. They were interviewed to determine whether they met the study prerequisites. The exclusion criteria were as follows: smoking more than 10 cigarettes per day (following Kirschbaum et al., 1993b, 1994); abuse of alcohol (no more than 20 g/days for females and 30 g/day for males) or other drugs; having been under general anesthesia in the past 3 months; having experienced a stressful life event in the past year (e.g., death of a relative, divorce, or separation, having been fired, severe personal illness, serious personal accident or injury, serious illness in the family); presenting severe sight or hearing problems or a neurological, cardiovascular, psychiatric, endocrine, or HPA axis disease; or using drugs that can affect cognitive or emotional functioning or hormone levels (e.g., glucocorticoids, antidiabetics, antidepressants, anticoagulants, β-blockers, benzodiazepines, or hypnotics). All the females had their last period at least 2 years before the study, and none were receiving hormone replacement therapy.

The final sample consisted of 66 participants (53% female) between 56 and 75 years old (see Table 1), with a medium subjective socioeconomic status (measured using the nine-rung ‘social ladder’, cf., Adler and Stewart, 2007; SES; where 1 was the lowest education and income and the worst jobs, and 10 was the best education, income, and jobs). Of all the participants, 53.1% had an educational level above secondary school, and 82.8% were retired. The mean body mass index was 26.522 (*SEM* = 0.531). The stress perceived by the participants during the previous month was low (*M* = 17.110, *SEM* = 0.732), according to the scores (ranging from 0 to 56). They were all non-smokers, except one male in the stress condition who smoked four cigarettes per day. Given that he met the criteria for inclusion in the study, and that the statistical conclusions did not change after excluding this participant, we kept him in the statistical analyses.

**Table 1:** Means and standard errors for the study measures, in the total sample and by experimental condition and sex.

**Variable**	**Total sample** **(*n =* 66)**	**Stress** **(*n =* 30)**	**Control** **(*n =* 36)**	***t(p)/ X^**2**^(p)***	**Males** **(*n =* 31)**	**Females** **(*n =* 35)**	***t(p) X^**2**^(p)***
Age (years)	64.24 (0.573)	65.07 (0.894)	63.56 (0.732)	−1.321 (0.191)	64.97 (704)	63.60 (0.878)	1.195 (0.236)
SES	5.49 (0.150)	5.40 (0.265)	5.58 (0.157)	0.583 (0.562)	5.32 (0.236)	5.63 (0.193)	– 1.019 (0.312)
BMI (kg/m^2^)	26.522 (0.531)	26.334 (0.762)	26.687 (0.749)	0.330 (0.743)	26.815 (0.780)	26.283 (0.732)	0.496 (0.622)
Educational level (%)				3.662 (0.599)			4.468 (0.484)
No studies	1.6	0	2.8		0	2.9	
Basic studies	20.3	26.7	13.9		10.3	28.6	
High school	25	23.3	25		27.6	22.9	
College or higher	53.1	49.9	53.2		62	45.8	
CD– Risc	30.85 (0.585)	30.73 (0.796)	30.94 (0.855)	0.177 (0.860)	30.87 (0.885)	30.83 (0.789)	0.032 (0.974)
Active coping	2.934 (0.054)	2.944 (0.088)	2.936 (0.067)	– 0.166 (0.868)	2.917 (0.076)	2.95 (0.077)	– 0.304 (0.762)
Emotional coping	2.722 (0.051)	2.644 (0.066)	2.788 (0.075)	1.415 (0.162)	2.658 (0.078)	2.776 (0.066)	−1.155 (0.253)
Avoidance coping	1.804 (0.045)	1.773 (0.070)	1.832 (0.059)	0.646 (0.521)	1.713 (0.055)	1.884 (0.067)	−1.917 (0.060)
Cognitive coping	2.972 (0.034)	2.975 (0.054)	2.969 (0.043)	−0.087 (0.931)	2.964 (0.051)	2.978 (0.045)	−0.215 (0.830)
PSS-14	17.11 (0.732)	16.10 (1.045)	17.97 (1.012)	1.282 (0.205)	17.83 (1.137)	16.49 (0.949)	0.917 (0.363)

*Data are presented for the total sample (N = 66), participants in the stress (n = 30) and control (n = 36) conditions, and males (n = 31) and females (n = 35). BMI, body mass index; CD-Risc, Connor-Davidson Resilience Scale; PSS, Perceived Stress Scale; SES, subjective socioeconomic status scale. Data represent means (standard errors), t(p) are presented for the differences between stress and control condition and for males and females. Data for educational level is presented in percentages, and X^2^(p) are presented for the differences between stress and control condition and for males and females*.

The study was designed and carried out in accordance with the Declaration of Helsinki, and the experimental protocol was approved by the university's Ethics Committee. Participants were informed both verbally and in writing about the study content and the measures that would be taken, and each participant gave his/her written informed consent before participating in the study. To avoid anticipatory responses, they were not informed about the stress task until they received the instructions.

### Procedure

Participants were invited to take part in a 2-h experimental session. They were randomly assigned to the stress (14 males and 16 females) or control (17 males and 19 females) condition. To control the circadian rhythm of cortisol secretion and sex differences in the cortisol response to stress (Allen et al., 2014; Pulopulos et al., 2018), the sessions were held in the afternoon (16–18 h or 18–20 h), and both the time when the participants started the session and sex were counterbalanced across conditions.

**Figure 1:**
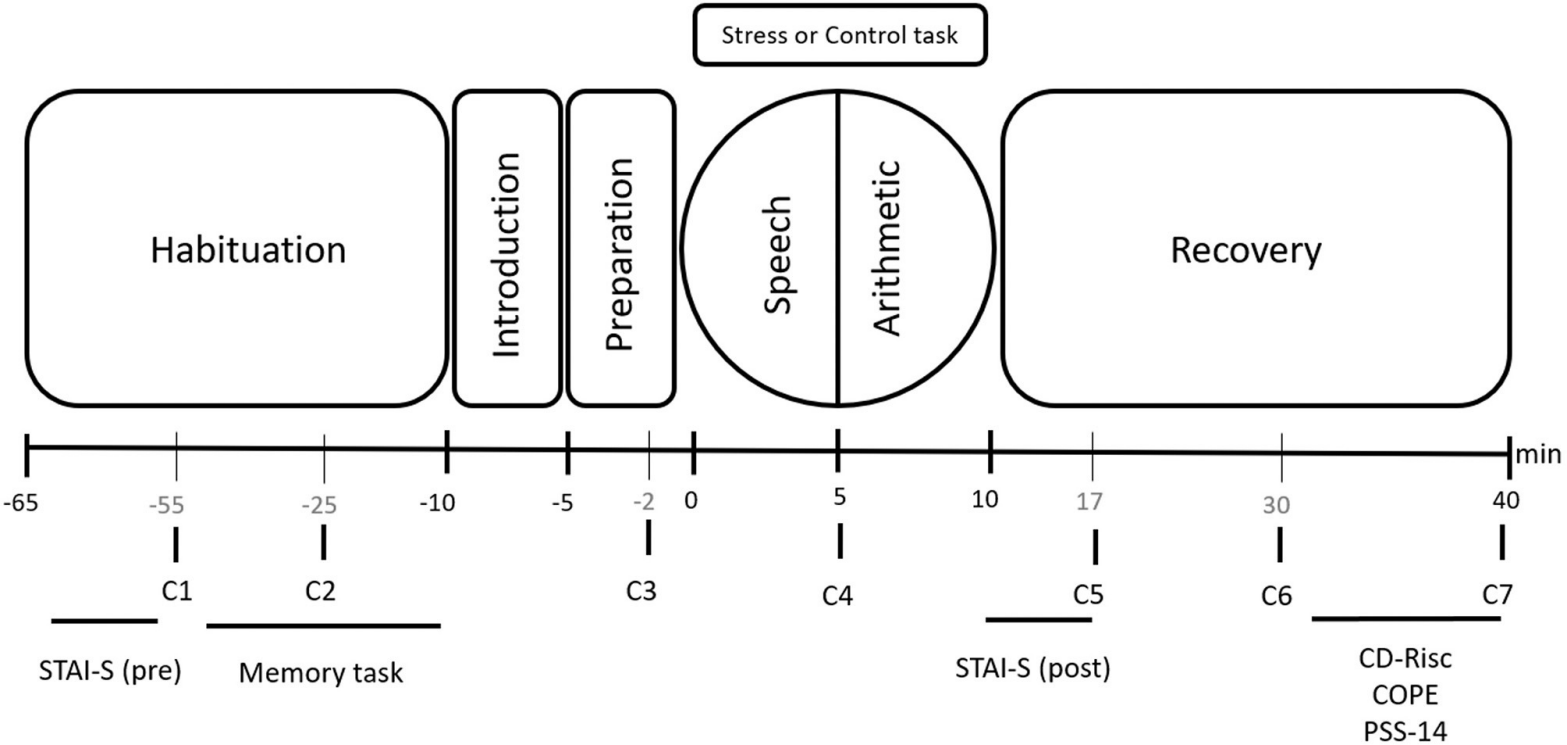
Timeline of the experimental session. Salivary cortisol samples: C1, C2, C3, C4, C5, C6, and C7. Psychological tests: State anxiety (STAI-S, pre and post); Resilience (CD-Risc); Coping strategies (COPE); and Perceived stress (PSS-14).

Participants were asked to sleep their usual number of hours, avoid intense physical exercise and the consumption of alcoholic drinks for 1 day prior to the study, and not eat or drink, smoke, or consume any type of stimulant (e.g., caffeine) for 2 h prior to the study. When participants arrived at the laboratory, the experimenter verified that they had followed these instructions. After consenting to take part in the study, participants had 55 min of habituation before performing the task. During the first 10 min, participants completed the state anxiety subscale of the State-Trait Anxiety Inventory (STAI), in order to obtain a baseline measure of their state anxiety (STAI-S pre). Then, the first saliva sample (C1) was taken 10 min after the beginning of this habituation phase to obtain the basal cortisol level. This was followed by completion of the memory task, which is not part of the current research question and consisted of viewing 60 emotional and neutral images extracted from the International Affective Picture System (IAPS; Lang et al., 2005). In the middle of this task, participants provided the second saliva sample (C2). Next, participants received instructions for the stress or control task. Participants were then informed that they had 5 min to prepare a speech (preparation phase). They provided the third saliva sample (C3) while preparing the speech. Between the Speech and Arithmetic tasks, the fourth saliva sample was collected (C4). After the stress or control task, participants were asked to remain calm for 30 min. During this period, they completed the post-task state anxiety subscale (STAI-S post) and provided the last three saliva samples (C5, C6, and C7). Finally, during the last 10 min, participants filled out the remaining questionnaires [perceived stress, coping styles, and resilience (Figure 1)], following the procedure described by Zoccola et al. (2010).

### Stress and Control Tasks

The TSST (Kirschbaum et al., 1993a) was used to provoke psychological and cortisol responses to stress in the stress condition. The TSST consists of two tasks that last 5 min each: a free speech task (job interview) and an arithmetic task. During both tasks, participants had to stand at a distance of 1.5 m from a committee composed of a woman and a man. Similar to our previous studies, in order to control for other confounding variables, a member of the committee who was of the opposite sex to the participant carried out all the interactions, which also elicits increased levels of cortisol (Duchesne et al., 2012), anxiety, and discomfort (Martinson and Zerface, 1970; Dodge et al., 1987; McCubbin et al., 1991; Chorney and Morris, 2008). In addition, both tasks were recorded using a video camera and a microphone, both of which were visible to the participant. Several previous studies have shown that this task provokes robust psychological and endocrine responses to stress in young and older people (for a review, see: Pulopulos et al., 2018).

Salivary samples were collected before the introduction to the TSST during the habituation phase (−55 and −25 min pre-stress), during preparation for the TSST (−2 min pre-stress), immediately after the speech (+5 min), and after the TSST (+17, +30, and +40 min). Measurements of state anxiety were taken during the habituation and the post-task phases.

Participants assigned to the control condition performed a non-stressful task with similar mental workload and physical effort to the TSST, but without stressful components (Dickerson and Kemeny, 2004) such as social evaluation and lack of control. The control task consisted of 5 min of reading aloud, followed by 5 min of counting backward by fives (as previously used in Almela et al., 2011; Hidalgo et al., 2012). The control task was not performed in front of an audience. Saliva sampling, questionnaire administration, and phase durations were similar to those of the stress condition.

### Measures

#### Stress Response

##### Salivary Cortisol

Throughout the experimental session, participants provided seven saliva samples with Salivettes (SARSTEDT, Nümbrecht, Germany). After the samples were collected, they were centrifuged at 4,000 rpm for 15 min to obtain a transparent supernatant that was stored at −80°C until the analyses were carried out. The cortisol concentrations were determined using the Salimetrics commercial salivary cortisol enzyme-linked immunosorbent assay kit (Newmarket, UK). The sensitivity of the assay was 0.007 μg/dL, and the intra- and inter-assay coefficients of variation were all below 10%. The samples from each subject were analyzed in the same assay and in duplicate.

##### Anxiety State

It was evaluated using the Spanish version (Seisdedos, 1988) of the STAI-S (Spielberger et al., 1970). Participants had to rate how they felt in general at the time of completing the questionnaire. The STAI-S consists of 20 items answered on a Likert scale ranging from 0 (“nothing”) to 3 (“a lot”). Cronbach's alpha for this study was α = 0.90.

#### Perceived Stress

To evaluate perceived stress, we used the Spanish version (Remor, 2006) of the Perceived Stress Scale (Cohen et al., 1983). The Perceived Stress Scale is a self-report questionnaire that assesses perceived stress in the past month. We intended to determine whether there were differences in perceived stress between groups before they performed the TSST or control tasks. This scale consists of 14 items with a 5-point response format (from 0 “never” to 4 “very often”), with 56 being the highest score and 0 the lowest. The higher the score, the greater the perceived stress. The Cronbach's alpha for this questionnaire in the current sample was 0.70.

#### Resilience

Resilience was assessed using the short version (Campbell-Sills and Stein, 2007) of the Connor-Davidson Resilience Scale (CD-Risc) (Connor and Davidson, 2003). The 10-item CD-Risc scale assesses one's ability to evaluate and face adversity and stress during the past month (e.g., able to adapt to change, face adversities, and bounce back, and belief that one can deal with whatever comes). The Spanish version of the scale (Notario-Pacheco et al., 2011) consists of 10 items rated on a 5-point Likert scale (0 = “rarely”, 4 = “almost always”). The results obtained range from 0 to 40, and higher scores indicate higher levels of resilience. The CD-Risc is considered a good measure of resilience, given that it has been related to lower post-traumatic symptoms, increased social support, active coping strategies, and quality of life, less perceived stress and depression, and fewer avoidant coping strategies (Serrano-Parra et al., 2013; Thompson et al., 2018). Cronbach's alpha for this study was α = 0.80.

#### Coping Strategies

Coping strategies were examined using the Spanish version of the Coping Orientations to Problems Experienced Inventory (COPE; Crespo and Cruzado, 1997). The COPE is a self-report questionnaire on which subjects have to indicate what they usually feel and do in a stressful situation. The items are rated on a 4-point Likert scale from 1 (“I do not usually do it”) to 4 (“I usually do this a lot”). The questionnaire consists of 60 items grouped into 15 subscales (social support, religion, humor, alcohol or drugs, planning and active coping, avoiding coping, focusing on emotions, acceptance, denial, restraint coping, concentrating efforts to solve the situation, personal growth, positive reinterpretation, behavioral disengagement, and escape). These 15 subscales can be grouped into second-order factors with a four-factor structure that includes active, cognitive, and emotional coping (i.e., active coping strategies), and avoidance (i.e., passive coping strategies) (Carver et al., 1989; Hasking and Oei, 2002). Because the majority of the studies have related resilience to active and avoidance coping (for review, see Southwick et al., 2005), we used these two second-order factors to determine whether resilience leads to more adaptive and less maladaptive coping strategies. We employed the active (planning, active coping, and suppression of competing activities) and avoidance (behavioral disengagement, mental disengagement, denial, and religious coping) factors. In our data, Cronbach's alpha for all the items was α = 0.74. Cronbach's alphas for active coping and avoidance coping were α = 0.76 and α = 0.70, respectively.

There is no consensus about the relationship between resilience and emotional coping. This may be due to the nature of the scale itself, which includes different responses ranging from the adaptive mobilization of sources (i.e., seeking emotional support) to the expression of negative emotions (i.e., venting emotions), suggesting that emotional coping is not essentially adaptive or maladaptive (Carver and Connor-Smith, 2010). In the current study, Cronbach's alpha for emotional coping was α = 0.75; however, cognitive coping showed a low Cronbach's alpha that was not adequate (α = 0.42). In addition, the analyses carried out with these factors confirmed they do not mediate the relationship between resilience and stress outcomes (cortisol and anxiety). Therefore, these results have not been reported.

### Data Management and Statistical Analysis

Cortisol values were not normally distributed and, therefore, were log transformed. Cortisol reactivity was calculated by subtracting pre-stress levels (mean −55 min, −25 min, and −2 min) from the highest cortisol indexes reached for each participant. Anxiety reactivity was calculated as the difference between the pre- and post-task measures for each condition (Almela et al., 2012; Villada et al., 2014a, 2018). The area under the curve with respect to the increase (AUCi) for cortisol values was computed as an index of the cortisol response to TSST (for the formula see: Pruessner et al., 2003).

The Student's *t*-test for independent samples and the *X*^2^ test were performed to evaluate the differences between groups in psychological variables and educational level, respectively.

A mixed analysis of variance (ANOVA) was used to investigate the differences between conditions in the changes in cortisol and anxiety levels during the session, with time (cortisol: −55, −25, −2, +5, +17, +30, +40 min; anxiety: pre-task vs. post-task) as the within-subject factor and condition (stress vs. control) and sex (males vs. females) as the between-subject factors. The statistical conclusion remains the same if the analyses are repeated using mixed-effects regression. Two-way ANOVAs were used to study condition and sex differences in cortisol reactivity and AUCi indexes. Pearson correlations were used to investigate the relationship between resilience and delta changes in cortisol and anxiety.

Following Preacher et al. (2007), we performed moderated mediation analyses to investigate whether coping strategies mediate the relationship between resilience and cortisol and anxiety responses in the stress condition. It is generally agreed that there should only be one requirement to establish mediation: the indirect effect (a^*^b) has to be significant (Zhao et al., 2010; Hayes, 2017). For cortisol, we entered resilience as the independent variable, active coping as the mediator variable, cortisol reactivity as the dependent variable, and condition (stress or control) as the moderator variable. Standardized values were used to perform the moderated mediation analysis. Bias-corrected bootstrapping was conducted to assess the mediating effect of active coping on the relationship between resilience and cortisol reactivity. We also observed the moderator effect of the condition in the relationships between resilience and cortisol reactivity and between active coping and cortisol reactivity. The same methods were employed with avoidance coping as a mediator. When anxiety reactivity was examined as the dependent variable, we carried out the same analyses, with active and avoidance coping as mediators and condition (stress or control) as moderator. Bootstrap data resampling procedures establish confidence intervals (CIs) to test the statistical significance of an indirect effect (Shrout and Bolger, 2002). Confidence intervals are considered statistically significant when they do not include zero. The analysis was based on 10,000 bootstrap iterations, and the CI was set at 95%, as recommended by Mallinckrodt et al. (2006). *Post-hoc* power analysis showed that all the relationships included in the mediations presented an adequate power >0.80, with an alpha level of *p* = 0.05. Only the relationship between resilience and avoidance coping showed a statistical power of 0.247. However, these power analyses are based on linear regression analyses. Although the sample size can be considered relatively small, the bootstrap technique draws random samples of a fixed sample size with replacement from the dataset, which increases the statistical power. This type of statistical approach takes the real sample size into consideration and controls for this factor in the analyses (Hayes, 2017). Therefore, the use of bootstrap-corrected confidence intervals solves the issues of a relatively small sample size. We used Hayes' PROCESS macro (Hayes, 2017), specifically model number 15, with SPSS (version 26; IBM Corporation, Armonk, NY, USA). One outlier in the cortisol data (one male in the control condition) and four outliers in the anxiety data (two females: one in the stress condition and one in the control condition; two males: one in the stress condition and one in the control condition) were winsorized by replacing extreme values that differed by more than three standard deviations (SD) from the mean with the value corresponding to ±3 SD. No differences were found in the analyses of cortisol, anxiety, or moderated mediations after excluding the outliers. The different numbers of participants included in the analyses of cortisol and the psychological variables are explained by missing values.

*Post-hoc* planned comparisons were performed using Bonferroni adjustments for the *p-*values. The level of significance was set at 0.05. When not otherwise specified, the results are presented as means ± SEM. All statistical analyses were performed with SPSS 26.0. For an easy interpretation of the figures, the values shown represent raw values and not log-transformed values.

## Results

### Preliminary Results

#### Sample Characteristics

Table 1 shows sample characteristics. No significant differences between conditions were found for age (*t* = −1.321, *p* = 0.191), SES (*t* = 0.583, *p* = 0.562), BMI (*t* = 0.330, *p* = 0.743), educational level (*X*^2^ = 3.662, *p* = 0.599), resilience (*t* = 0.177, *p* = 0.860), coping strategies (all *p* > 0.05), or perceived stress (*t* = 1.282, *p* = 0.205).

#### Cortisol Response^1^

**Figure 2:**
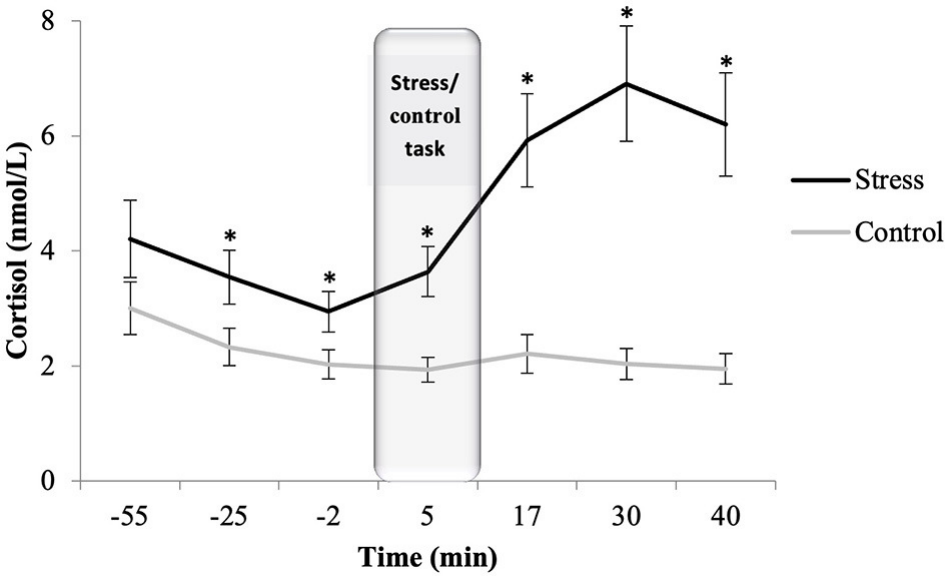
Salivary cortisol concentrations for stress (*n* = 27) and control (*n* = 32) conditions. Depicted values are means and error bars represent the SEM of raw cortisol values (*p < 0.020).

The mixed ANOVA showed effects of time [*F*_(1.99,109.92)_ = 9.884, *p* < 0.001, $\eta_{\text{ p}}^{2}$ = 0.152], sex [*F*_(1,55)_ = 9.140, *p* = 0.004, $\eta_{\text{ p}}^{2}$ = 0.143], condition [*F*_(1,55)_ = 21.299, *p* < 0.001, $\eta_{\text{ p}}^{2}$ = 0.279], and the time and condition interaction [*F*_(1.99,109.92)_ = 17.092, *p* < 0.001, $\eta_{\text{ p}}^{2}$ = 0.237]. *Post-hoc* analyses revealed that cortisol levels were higher in males than in females. Specifically, cortisol levels were significantly higher in the stress condition than in the control condition in all the samples (all *p* < 0.020), except −55 min (*p* = 0.087). In the stress condition, there were no significant differences between the first four salivary samples (all *p* > 0.100). However, cortisol levels were significantly higher after the stress task than during the stress task (+5 min vs. +17 min: *p* < 0.001). After peaking (+30 min), cortisol concentrations decreased until they showed no statistically significant differences from those of the habituation period (+40 min vs. −55 min*: p* = 0.235). In the control condition, there was a significant decrease in cortisol levels from the −55-min to −25-min saliva samples (*p* = 0.003). No other differences in cortisol levels were observed during the control session (all *p* > 0.05) (see Figure 2).

There were also differences in cortisol reactivity between conditions (*p* < 0.01). Participants in the stress condition demonstrated a response to the task, whereas those in the control condition did not (stress: M = 0.250, SEM = 0.044; control: M = *p* > 0.007, SEM = 0.038). Cortisol differences between conditions were also found in the AUCi [*F*_(1,58)_ = 15.102, η^2^_p_ = 0.207, *p* < 0.001]; participants in the stress condition showed higher AUCi values than participants in the control condition (*p* < 0.001). No differences were found between sexes in AUCi or cortisol reactivity levels (all *p* > 0.500).

#### Anxiety Response

**Figure 3:**
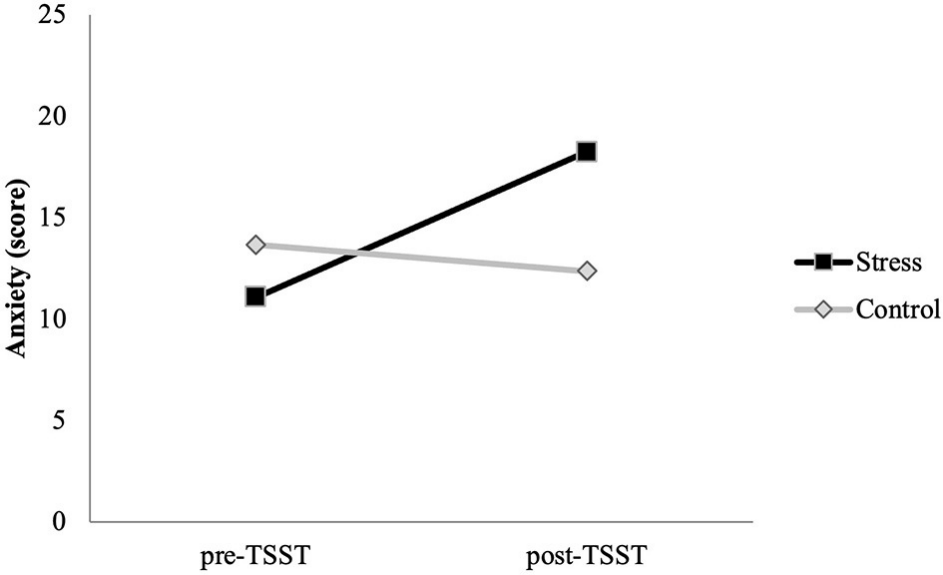
Pre- and post-task anxiety for stress (*n* = 30) and control (*n* = 36) conditions.

The mixed ANOVA showed significant effects of time [*F*_(1,62)_ = 19.005, *p* < 0.001, $\eta_{\text{ p}}^{2}$ = 0.235] and the time × condition interaction [*F*_(1,62)_ = 38.123, *p* < 0.001, $\eta_{\text{ p}}^{2}$ = 0.381]. No baseline differences between conditions were found (*p* = 0.098). However, participants in the stress condition showed higher levels of anxiety after the task than participants in the control condition (*p* = 0.001). Moreover, participants in the stress condition showed higher levels of anxiety after the task than before it (*p* < 0.001). Anxiety levels in the control participants did not change significantly (*p* = 0.183) (Figure 3).

### Role of Resilience in Psychobiological Stress Response

#### Relationship Between Resilience and Stress Indicators

Pearson correlations showed no significant relationships between resilience and cortisol reactivity (stress: *r* = 0.226, *p* > 0.05; control: *r* = −0.101, *p* > 0.05) or anxiety reactivity (stress: *r* = −0.034, *p* > 0.05; control: *r* = −0.152, *p* > 0.05) in either condition.

#### Testing the Moderated Mediation Model: Cortisol Response

Active coping, understood as a second-order factor, was tested as a mediator in the association between resilience and cortisol reactivity. Moderated mediation analysis showed that higher resilience was associated with higher active coping (path *a*: B = 0.294, SE = 0.122, *p* = 0.019).

With regard to the moderating effect of condition in the relationship between resilience and cortisol reactivity and between active coping and cortisol reactivity, we observed that the interaction effects between resilience and the stress condition (*p* = 0.029) and between active coping and the stress condition (*p* = 0.009) were significant.

**Figure 4:**
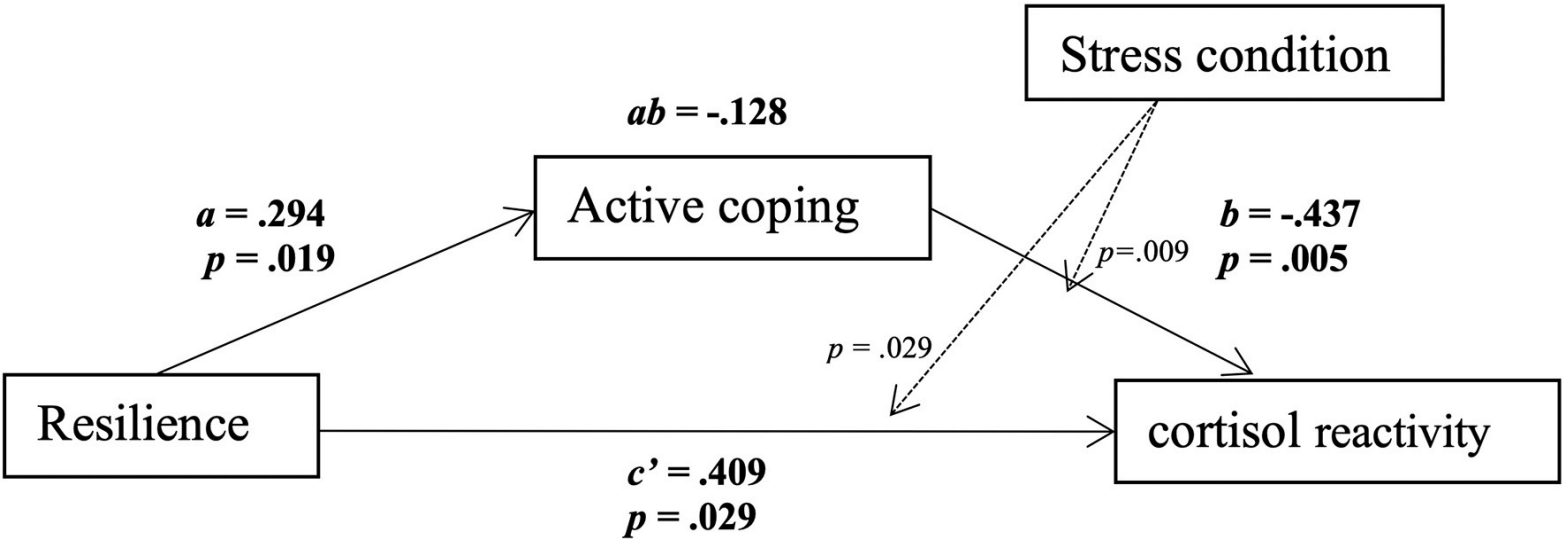
Moderation and mediation analysis with stress condition, using bias-corrected bootstrapping in conjunction with multiple regression analysis. Numbers on the lines show B and *p*-values. Solid lines indicate direct and indirect effects; dashed lines indicate moderations. Results are reported only for the stress condition. Resilience was positively related to active coping (path *a*: B = 0.294, SE = 0.122, *t* = 2.401, *p* = 0.019). Stress condition moderated the relationship between active coping and cortisol reactivity (active coping x condition: *p* = 0.009), and it also moderated the relationship between resilience and cortisol reactivity (resilience x condition: *p* = 0.029). The relationship between active coping and cortisol reactivity was significant for the stress condition (path *b*: B = −0.437, SE = 0.151, *t* = −2.891, *p* = 0.005). The conditional direct effect of the relationship between resilience and cortisol reactivity was also significant for the stress condition (path *c'*: *B* = 0.409, SE = 0.182, *p* = 0.029, 95% confidence interval = 0.043 to 0.775). There was an indirect effect of resilience on cortisol reactivity through active coping in the stress condition (path *ab*: *B* = −0.128, 95% confidence interval = −0.329 to −0.008).

The relationship between active coping and cortisol reactivity was negative and significant in the stress condition (path *b*: B = −0.437, SE = 0.151, *t* = −2.891, *p* = 0.005), but not in the control condition (path *b*: B = 0.165, SE = 0.163, *t* = 1.008, *p* = 0.317). Analysis of the conditional direct effect of the relationship between resilience and cortisol reactivity, controlling for active coping, showed a significant direct effect in the stress condition (path *c'*: *B* = 0.409, SE = 0.182, *p* = 0.029), but not in the control condition (path *c'*: *B* = −0.103, SE = 0.139, *p* = 0.460). The conditional indirect effect of resilience on cortisol reactivity through active coping was examined for both conditions. The results showed an indirect effect of resilience on cortisol reactivity through active coping in the stress condition (path *ab*: *B* = −0.128, 95% CI = −0.329 to −0.008), but not in the control condition (path *ab*: *B* = 0.048, 95% CI = −0.013 to 0.152) (Figure 4).

Regarding the moderated mediation analysis between resilience and cortisol reactivity through avoidance coping, Table 2 shows that the relationship between resilience and avoidance coping was not significant (path *a*: B = −0.055, SE = 0.130, *p* = 0.672). Moreover, neither the interaction effect between resilience and the stress condition (*p* = 0.268) nor the interaction effect between avoidance coping and the stress condition (*p* = 0.461) was significant. Analysis of the conditional direct effect of the relationship between resilience and cortisol reactivity, controlling for avoidance coping, did not show a significant direct effect in the stress condition (path *c'*: *B* = 0.231, SE = 0.188, *p* = 0.225) or the control condition (path *c'*: *B* = −0.030, SE = 0.139, *p* = 0.828). Finally, the conditional indirect effect of resilience on cortisol reactivity through avoidance coping was not significant in the stress condition (path *ab*: *B* = −0.016, 95% CI = −0.117 to 0.086) or the control condition (path *ab*: *B* = −0.007, 95% CI = −0.066 to 0.019). The findings observed with cortisol reactivity are not replicated with AUCi. However, we would like to note that this is not uncommon in cortisol research because these two indexes reflect different information (see Pulopulos et al., 2018, 2020).

**Table 2:** Moderated mediation between resilience and cortisol reactivity through avoidance coping.

**Dependent variable (Y): cortisol reactivity** **Mediator (M): Avoidance coping** **Moderator (W): Condition**							
		**Effect**	**SE**	***t***	***p***	**LLCI**	**ULCI**
a		−0.055	0.130	−0.426	0.672	−0.316	0.205
c'	Stress	0.231	0.188	1.227	0.225	−0.146	0.609
	Control	−0.030	0.139	−0.219	0.828	−0.308	0.247
ab	Stress	−0.016	0.047			−0.117	0.086
	Control	−0.007	0.022			−0.066	0.019

*Letters represent the relationship between resilience and avoidance coping (a), the direct effect (c'), and the indirect effect (ab) for each condition. LLCI, lower level of confidence interval; SE, standard error; UCLI, upper level of confidence interval*.

#### Testing the Moderated Mediation Model: Anxiety Response

Table 3 presents the results of the moderated mediation analysis of resilience and anxiety reactivity through active coping. The relationship between resilience and active coping was significant (path *a*: B = 0.343, SE = 0.118, *p* = 0.005). However, neither the interaction effect between resilience and the stress condition (*p* = 0.573) nor the interaction effect between active coping and the stress condition (*p* = 0.346) was significant. Therefore, analysis of the conditional direct effect of the relationship between resilience and anxiety reactivity, controlling for active coping, did not show a significant direct effect in the stress condition (path *c'*: *B* = 0.049, SE = 0.144, *p* = 0.734) or the control condition (path *c'*: *B* = −0.055, SE = 0.116, *p* = 0.633). Finally, the conditional indirect effect of resilience on anxiety reactivity through active coping was not significant in the stress condition (path *ab*: *B* = −0.085, 95% CI = −0.275 to 0.017) or the control condition (path *ab*: *B* = −0.026, 95% CI = −0.112 to 0.045). As Table 3 indicates, the moderated mediation analysis of resilience and anxiety reactivity through avoidance coping showed that the relationship between resilience and avoidance coping was not significant (path *a*: B = −0.024, SE = 0.126, *p* = 0.847). Moreover, neither the interaction effect between resilience and the stress condition (*p* = 0.933) nor the interaction effect between avoidance coping and the stress condition (*p* = 0.942) was significant. Therefore, the conditional direct effect of the relationship between resilience and anxiety reactivity, controlling for avoidance coping, did not show a significant direct effect in the stress condition (path *c'*: *B* = 0.057, SE = 0.144, *p* = 0.694) or the control condition (path *c'*: *B* = −0.072, SE = 0.115, *p* = 0.531). Moreover, the conditional indirect effect of resilience on anxiety reactivity through avoidance coping was not significant in the stress condition (ab: *B* = −0.001, 95% CI = −0.032 to 0.033) or the control condition (ab: *B* = −0.001, 95% CI = −0.034 to 0.028).

**Table 3:** Moderated mediation between resilience and anxiety reactivity through active or avoidance coping.

**Dependent variable (Y): anxiety reactivity** **Mediator (M): Active coping** **Moderator (W): Condition**							
		**Effect**	**SE**	***t***	***p***	**LLCI**	**ULCI**
a		0.343	0.118	2.898	0.005	0.106	0.579
c'	Stress	0.049	0.144	0.342	0.734	−0.240	0.339
	Control	−0.055	0.116	−0.479	0.633	−0.287	0.176
ab	Stress	−0.085	0.074			−0.275	0.017
	Control	−0.026	0.034			−0.112	0.045
**Dependent variable (Y): anxiety reactivity** **Mediator (M): Avoidance coping** **Moderator (W): Condition**							
		**Effect**	**SE**	***t***	***p***	**LLCI**	**ULCI**
a		−0.024	0.126	−0.194	0.847	−0.276	0.227
c'	Stress	0.057	0.144	−0.394	0.694	−0.345	0.232
	Control	−0.072	0.115	−0.628	0.531	−0.302	0.158
ab	Stress	−0.001	0.015			−0.032	0.033
	Control	−0.001	0.014			−0.034	0.028

*Letters represent the relationship between resilience and active or avoidance coping (a), the direct effect (c'), and the indirect effect (ab) for each condition. LLCI, lower level of confidence interval; SE, standard error; UCLI, upper level of confidence interval*.

## Discussion

The general purpose of the present study was to examine the role of resilience in health and well-being in older people by analyzing several components of their stress response. First, we investigated how resilience is related to the psychobiological response to an acute psychosocial stressor (i.e., the TSST). Second, we examined the mediating role of coping strategies in the relationship between resilience and the endocrine and psychological responses to this stressor. Our results showed that resilience was not directly related to cortisol or anxiety responses to the stressor. However, active coping strategies mediated resilience's relationship with cortisol, but not with anxiety. In contrast, passive coping strategies did not mediate the relationship between resilience and psychobiological components of the stress response. The current findings highlight the importance of resilience and coping in the regulation of the stress response, suggesting that these are factors that may prevent the development of stress-related pathologies associated with aging and facilitate healthy and satisfactory aging. Following the allostatic load model, the benefits of resilience and active coping on health could be due to a better regulation of the HPA axis in these people, as observed in this study. It should be noted that, although some participants in our sample were relatively young (the ages ranged from 56 to 75, and the mean age was nearly 65 years), our results are consistent with those from studies employing older samples (Hildon et al., 2009; Tomás et al., 2012).

### Resilience and the Psychobiological Response

In agreement with previous findings in older people, the stress task used in our study provoked an acute increase in cortisol and anxiety levels, compared to the control task (for reviews, see Allen et al., 2014; Pulopulos et al., 2018). Although there is an existing relationship between increased general fat tissue and altered HPA axis (Champaneri et al., 2013), little is known about the relationship between BMI and cortisol stress reactivity (Therrien et al., 2010). We did not find an association between BMI and cortisol reactivity in the stress nor in the control group. These results are in line with other studies, which do not find differences between obese and non-obese participants in the cortisol reactivity to the TSST in people aged from 50 to 70 years old (Jayasinghe et al., 2014) and in young people (Therrien et al., 2010; Herhaus and Petrowski, 2018; but see Cano-López et al., 2019 as an exception), suggesting that an increase in body mass is not associated to cortisol reactivity to an acute stressor. Moreover, contrary to our hypothesis, resilience was not correlated with cortisol or anxiety responses to the stressor. Two previous studies found that highly resilient individuals had lower overall cortisol secretion during acute stress than their less resilient peers (Mikolajczak et al., 2008; Ruiz-Robledillo et al., 2016). However, two other studies found that, consistent with our findings, resilience was not related to cortisol reactivity to the TSST (Simeon et al., 2007; García-León et al., 2019). With regard to anxiety, some studies found that resilience was associated with fewer anxiety symptoms (Hjemdal et al., 2010; Shi et al., 2015) and attenuated anxiety after a stressor (Ruiz-Robledillo et al., 2016), whereas others only found this association in females (Carvalho et al., 2016). Nevertheless, in our study, resilience was analyzed as a continuous variable, with no differentiation between individuals with high and low resilience. In addition, past research has focused on young people and people in chronic stress conditions, such as caregivers of people with autism, patients with cardiovascular diseases, and medical students, which could already have an altered HPA response. It has been found that whereas acute stress causes transient effects on the HPA response, chronic stress produces prolonged HPA activity, which led to an impaired negative feedback and both high and low long-term cortisol levels (Miller et al., 2007; Marković et al., 2011). Our results suggest that resilience alone is not related to the psychobiological response to an acute stressor in healthy older people.

### The Mediating Role of Active Coping Strategies in the Relationship Between Resilience and Cortisol

Despite failing to confirm our first hypothesis, we found that coping plays an important role in the relationship between resilience and stress regulation. It has been suggested that resilience is related to active coping strategies, which, in turn, influence well-being (Tomás et al., 2012; Smith et al., 2015). In our study, we observed that resilience was positively related to active coping, and that more active coping led to lower cortisol reactivity (i.e., partial mediation). Moreover, resilience was positively related to cortisol reactivity when controlling for active coping, but only in the stress condition. These results suggest that the relationships among resilience, coping, and stress reactivity may be explained by a competitive mediation [i.e., with a mediated effect (ab) and a direct effect (c) both existing and pointing in different directions; Zhao et al., 2010]. The positive direct association between resilience and cortisol reactivity suggests the possible existence of secondary mediators that we did not examine. This positive direct association indicates that in future mediations, the sign of the indirect mediation will be positive. That is, resilience will be positively related to the mediator, and the mediator will be positively related to cortisol reactivity (Zhao et al., 2010). Future research should include other physiological variables in order to explore other resilience biomarkers. Heart rate variability is one possible biomarker which has been proposed as an objective measure of cognitive flexibility, the ability to adapt to stress, and resilience (for a review, see Perna et al., 2019).

It is also possible that only resilient individuals who use active (adaptive) coping strategies are able to have a more adaptive stress response (Gloria and Steinhardt, 2014). Our finding showing a positive relationship between resilience and cortisol reactivity after controlling for active coping might support this idea. This relationship was not found in the previous correlations when we did not control for active coping. These results are consistent with studies showing that resilience is positively related to active coping strategies (Tomás et al., 2012; Mayordomo et al., 2016; Thompson et al., 2018), and that active coping has a positive influence on the physiological regulatory functions in situations of stress (Villada et al., 2017). Together, following Lazarus and Folkman model (1984) the current evidence suggests that resilience enhances the use of effective coping strategies as a resource to manage stress (Connor and Davidson, 2003), and it supports the idea that resilience and coping strategies are different constructs. In this line, according to the broaden-and-build theory of positive emotions (Fredrickson, 1998), resilient individuals may be aware of the benefits of positive emotions in stressful situations, appraising the situation positively and developing more effective and adaptive strategies to manage stress (Feder et al., 2009; Gloria and Steinhardt, 2014), leading them to deal with and recover from these situations more easily (Tugade and Fredrickson, 2004).

We can also speculate that these findings may reflect the neurobiological processes underlying resilience. Studies have shown that, in resilient individuals, greater gray matter volumes in the ventral medial prefrontal cortex, the rostral anterior cingulate cortex (ACC), and the subgenual ACC modulate the emotional responsiveness of the amygdala and its subsequent effective stress response (Feder et al., 2009; van der Werff et al., 2013). More precisely, the cortical thickness of the ACC has been positively associated with resilience and positive coping styles (Gupta et al., 2016; Holz et al., 2016). Therefore, the cortical thickness and volume of the ACC may be responsible for the feedback inhibition of the amygdala and, thus, explain individual differences in the extent and duration of stress circuit activations in resilient individuals (Carnevali et al., 2018). However, we did not measure the activity and connectivity of these areas, and so further studies are warranted.

### No Mediating Role of Active Coping Strategies in the Relationship Between Resilience and Anxiety

With regard to the anxiety response, and in contrast with our hypothesis, active coping did not mediate the relationship between resilience and the psychological response to a stressor. These results could be due to the lack of precision in our anxiety measure. The cortisol response is evaluated through seven measures during the entire stressful situation, whereas the anxiety response is only evaluated through two measures, before and after the stressful situation. Therefore, using only a pre- and post-anxiety measure could produce misleading results because emotional states have been found to change rapidly and interact with other emotional processes preceding the endocrine response, which is slower (Schlotz et al., 2008). Moreover, the baseline anxiety measure may not reflect a neutral anxiety state due to expectations about the experiment. Therefore, it would be informative to collect the measures while people experience stress (not before or after it) (Campbell and Ehlert, 2012). Additional explanations could be related to the concept of emotion regulation. In adults, emotion regulation increases with age (Charles and Piazza, 2009), and this capacity may facilitate control over their emotional arousal (Nielsen et al., 2008). Therefore, in the present study, anxiety reactivity in the participants in the stress condition might have been more dependent on their age and less influenced by resilience or active coping strategies. Overall, the mixed results obtained for the influence of resilience on physiological and psychological variables are consistent with psychoendocrinological studies that show no correspondence between physiological and affective responses to laboratory stress tasks (Campbell and Ehlert, 2012; Villada et al., 2014b). Our results suggest that physiological and psychological reactions to a stressor apparently work in different ways, with precise and different data collection being necessary in each case (Campbell and Ehlert, 2012).

### The Role of Avoidance Coping Strategies

Unexpectedly, in the present study, resilience did not predict avoidance coping strategies, and avoidance coping was not related to cortisol or anxiety reactivity. Although this result did not support our hypothesis, our findings agree with a recent study showing that avoidance coping was not associated with or dependent on resilience (Smith et al., 2015). One explanation could be that a decrease in passive (maladaptive) coping strategies (i.e., mental disengagement and denial) would influence the way the individual evaluates the event and have a growing influence on resilience (Gloria and Steinhardt, 2014), and perhaps, on his or her engagement in active coping strategies (Thompson et al., 2018). Therefore, less use of maladaptive coping strategies would predict resilience, in contrast to our primary explanation that resilience triggers less use of maladaptive strategies, which was our hypothesis. As explained above, resilient individuals seem to experience positive emotions when a stressful situation occurs, rather than negative emotions, as in avoidance coping (Mayordomo et al., 2016). Thus, the fact that resilient people tend to exhibit lower levels of denial and experience more positive emotions during stressful situations triggers an upward spiral toward active coping and enhanced well-being (Fredrickson, 2001).

### Limitations

Despite this study's novel findings, some limitations should be considered. First, the results of this study should be replicated in larger samples with different ages and/or age-related diseases (i.e., diabetic or hypertensive older people) to ensure its generalization to the whole population given that our participants were selected based on their good physical and psychological health. Second, due to small sample size and in order to do not reduce the power of our statistical analyses, we did not study the role of sex on the relationships among resilience, coping, and cortisol. So, further studies are needed to investigate the influence of sex on these relationships. Third, the timing of the measurements is an important aspect to consider. In our study, we measured stable psychological aspects at the end of the session. Although no differences between conditions were observed with these measures, the stress task might have affected the responses. Future studies may benefit from asking the participants to complete the questionnaire on a different day and in non-stressful conditions. In addition, future research on the psychological response to stress exposure should add repeated real-time emotional reports, and not just pre- and post-task measures, in order to more adequately represent the stress experience. Finally, given the cross-sectional nature of this study, causality and directionality could not be determined.

## Conclusions and Future Directions

In conclusion, the results of this study suggest that greater resilience is associated with active coping strategies, which, in turn, are related to a lower cortisol response to stress in healthy older people. These results differ from those found for young individuals, in whom resilience was directly related to the psychological and physiological response to an acute stressor. Our results highlight the importance of the relationship between resilience and active coping strategies and are in line with other studies investigating this relationship in older samples. However, to the best of our knowledge, this is the first study to investigate the relationships between resilience, coping, and psychobiological factors such as cortisol and anxiety, using a laboratory-based stress paradigm in healthy older people.

Future research on stress protective factors should integrate resilience and coping into a theoretical framework where environmental and social support agents, such as community programmes or support groups, may play an important role in overcoming obstacles and enhancing personal growth (Fletcher and Sarkar, 2013; MacLeod et al., 2016). It is also important to investigate the moderating effects of resilience on other situational factors, such as adverse childhood experiences or chronic stressors (Connor et al., 2003). Overall, we encourage future studies to further examine the relationship between resilience and physiological and emotional stress responses in order to advance in this area. These studies would help to design interventions that consider the central role of resilience in the coping process and in overcoming stress-related pathologies, in an effort to improve the well-being of older people.

## Data Availability Statement

The raw data supporting the conclusions of this article will be made available by the authors, without undue reservation.

## Ethics Statement

The studies involving human participants were reviewed and approved by Universitat de València. The patients/participants provided their written informed consent to participate in this study.

## Author Contributions

MZ-F participated in the acquisition and interpretation of the data, managed the literature search, undertook the statistical analyses, and, with AS and VH, interpreted the results, revised the literature, and wrote the manuscript. IC-S participated in the data acquisition and interpretation of the data. MP controlled the acquisition of the data and revised the manuscript and the statistical analyses. All the authors contributed to and approved the final manuscript.

## Conflict of Interest

The authors declare that the research was conducted in the absence of any commercial or financial relationships that could be construed as a potential conflict of interest.

## Footnotes

^1^As BMI is clearly associated to HPA-axis functioning, we tested the relationship between the BMI and cortisol changes for the two conditions but no significant correlations were found (both *p* > 0.05).
